# A Novel, Synthetic, Neuroactive Steroid Is Effective at Decreasing Depression-Like Behaviors and Improving Maternal Care in Preclinical Models of Postpartum Depression

**DOI:** 10.3389/fendo.2018.00703

**Published:** 2018-11-23

**Authors:** Laverne Melón, Rebecca Hammond, Mike Lewis, Jamie Maguire

**Affiliations:** ^1^TEACRS Program, Tufts University School of Medicine, Boston, MA, United States; ^2^SAGE Therapeutics, Cambridge, MA, United States; ^3^Neuroscience Department, Tufts University School of Medicine, Boston, MA, United States

**Keywords:** neurosteroids, postpartum depression (PPD), HPA axis, benzodiazepine (BDZ), KCC2 = potassium chloride cotransporter 2

## Abstract

Preclinical testing of treatments for postpartum depression (PPD) has been limited due to the lack of available animal models of such a complex disorder. To address this limitation, our laboratory has generated unique preclinical mouse models that exhibit abnormal postpartum behaviors. Mice with a loss or reduction in the expression of the GABA_A_ receptor (GABA_A_R) δ subunit (*Gabrd*^−/−^ or *Gabrd*^+/−^, respectively) and mice that lack the K^+^/Cl^−^ co-transporter, KCC2, specifically in corticotropin-releasing hormone (CRH) neurons (KCC2/Crh mice) exhibit depression-like behaviors restricted to the postpartum period and deficits in maternal care, which serve as useful tools for testing novel therapeutic compounds. Utilizing these preclinical models, we tested the ability of a novel, synthetic, neuroactive steroid developed by SAGE Therapeutics, SGE-516, to improve abnormal postpartum behaviors. *Gabrd*^−/−^, *Gabrd*^+/−^, and KCC2/Crh dams treated with SGE-516 (450 mg/kg chow) during late pregnancy exhibit a decrease in depression-like behaviors and improvements in maternal care at 48 h postpartum. Interestingly, acute treatment with SGE-516 also exhibits robust therapeutic effects in these preclinical PPD models. We previously discovered abnormal stress reactivity associated with hypothalamic-pituitary-adrenal (HPA) axis dysregulation associated with depression-like behaviors in the preclinical PPD models, evident from an increase in stress-induced corticosterone levels and dephosphorylation and downregulation of KCC2 in the paraventricular nucleus of the hypothalamus (PVN) during the peripartum period. Here we demonstrated that SGE-516 treatment is sufficient to prevent the stress-induced increase in corticosterone and dephosphorylation and downregulation of KCC2 in the PVN. In contrast, and consistent with the distinct pharmacology of SGE-516 compared to benzodiazepines, treatment with clobazam (250 mg/kg chow) did not alter the depression-like phenotype or deficits in maternal care observed in these preclinical models of PPD. These findings are consistent with the positive double-blind, randomized, placebo-controlled trial findings of a similar compound, brexanolone, in the treatment of patients with postpartum depression. Further, these findings validate the use of these preclinical models of PPD for screening novel compounds for the treatment of postpartum depression.

## Introduction

Postpartum depression impacts nearly 20% of mothers (1, 2) and a much larger percentage (up to 75%) suffer from postpartum blues (3). Despite the high incidence of postpartum mood disorders, there has been a lack of research into the underlying biological mechanisms and potential treatments, in part, due to the lack of animal models required for preclinical research. Due to the time course of symptom presentation, the decline in ovarian hormones have been implicated in postpartum depression (4). However, women with postpartum depression do not exhibit differences in steroid hormone levels compared to controls [for review see (5)]. Interestingly, hormone withdrawal only induces depression symptoms in women with a history of postpartum depression (6), suggesting that they may be differentially sensitive to ovarian hormones. Women with postpartum depression do exhibit lower levels of allopregnanolone levels compared to healthy controls (7, 8), implicating neurosteroids and their site of action in postpartum depression. GABA_A_Rs are a principal target for neurosteroid action (9–12) [for review see (13, 14)], in particular extrasynaptic, GABA_A_R δ subunit containing receptors, which mediate tonic GABAergic inhibition and confer neurosteroid sensitivity (15). These receptors are unique from synaptic receptors, particularly those incorporating the GABA_A_R γ2 subunit, which are sensitive to benzodiazepines (15).

Our research previously demonstrated that mice that lack the GABA_A_R δ subunit, *Gabrd*^−/−^ mice, exhibit abnormal postpartum behaviors, including depression-like behaviors restricted to the postpartum period and deficits in maternal care (10). It was previously demonstrated that the abnormal maternal care is due to deficits in the dam, not the pup, through cross-fostering experiments (10). Utilizing this model, our laboratory was able to investigate potential mechanisms contributing to the abnormal postpartum phenotype in this model, which pointed to dysregulation of the physiological response to stress, mediated by the HPA axis, during the postpartum period (16).

Based on these findings, we explored the regulation of the HPA axis during the peripartum period and identified a role for the K^+^/Cl^−^ co-transporter, KCC2, which is required for effective GABAergic inhibition [for review see (17)], in the regulation of CRH neurons that control the body's physiological response to stress. The stress-induced activation of the HPA axis has been shown to involve compromised chloride homeostasis due to a dephosphorylation of KCC2 at residue Ser940 and downregulation of KCC2 in the PVN (18, 19). We previously demonstrated that the suppression of the HPA axis during the peripartum period involves maintenance of KCC2 expression in the PVN (20). To further examine the role of the HPA axis in contributing to vulnerability to mood disorders during the postpartum period, we generated mice that lack KCC2 specifically in CRH neurons (KCC2/Crh mice) (20). KCC2/Crh mice also exhibit depression-like behaviors and deficits in maternal care restricted to the postpartum period (20).

Here we utilize these preclinical PPD models to test the therapeutic effectiveness of GABA receptor modulators in the treatment of postpartum depression-like behaviors, specifically SGE-516, which is a novel, synthetic allopregnanolone analog, or clobazam, a benzodiazepine. Our data demonstrate that SGE-516 is effective in decreasing depression-like behaviors and improving maternal care in these preclinical PPD models. In contrast, the benzodiazepine, clobazam, is ineffective at altering abnormal postpartum behaviors in these preclinical PPD models. These results support a recent double-blind, randomized, placebo-controlled trial, demonstrating that brexanolone IV, a proprietary formulation of allopregnanolone, is effective in treating postpartum depression (21, 22) and validate the use of these preclinical models for assessing the effectiveness of potential treatments for PPD.

## Materials and methods

### Animals

Adult (>P60) female Wild Type (WT), *Gabrd*^−/−^, *Gabrd*^+/−−^, or KCC2/Crh mice were bred and housed at Tufts University School of Medicine's Division of Laboratory Animal Medicine facility under a 14/10 light schedule (lights on at 7:00 h) with *ad libitum* access to food and water. Littermates were used for all behavioral experiments. Females were harem bred with a male and the presence of a vaginal plug was used to time pregnancy (Positive plug = day 0). The estrous cycle was not monitored in virgin animals since we have determined that females are acyclic under our housing conditions, which entails using ventilated racks and isolation by sex (20). This is not abnormal or stress related given that is well-established that female mice become acyclic (anestrus) without close proximity to a male or exposure to pheromone in his urine (23, 24). Experiments were performed in virgin and postpartum (48–72 h) females. All procedures were approved by the Tufts University Institutional Animal Care and Use Committee and adhered to the ethical guidelines presented in the National Institutes of Health Guide for the Care and Use of Laboratory Animals (25).

Mice with a global knockout of the gene encoding the GABA_A_R δ subunit, *Gabrd*, (*Gabrd*^−/−^ mice) were originally obtained from Dr. Istvan Mody (University of California at Los Angeles, UCLA). The abnormal postpartum phenotype in this model was characterized in a previous manuscript (10). Mice lacking KCC2 specifically in CRH neurons were generated by crossing a floxed KCC2 (*KCC2*^*f*/*f*^) mouse line (a generous gift from Dr. Stephen J. Moss) with CRH-Cre mice originally obtained from the Mutant Mouse Regional Research Center (Stock # 030850-UCD). This mouse model, including the postpartum depression-like phenotype, has been thoroughly characterized in a recent manuscript (20). The KCC2/Crh mice are maintained on a 129/Sv background; whereas, the *Gabrd*^−/−^ mice are maintained on a C57Bl6/J background. Thus, these experiments assess the therapeutic effectiveness of compounds in ameliorating abnormal postpartum behavior in two different strains of mice.

### Drug treatments

Two different treatment strategies were employed in the current study, chronic administration of compounds that extends through late pregnancy and the early postpartum period and acute treatment exclusively during the postpartum period. Thus, the timing of these treatments will enable us to determine if treatment exclusively during the postpartum period is effective or if extended treatment is required. Chronic drug treatments were administered starting at day 14 of pregnancy and continuing until the end of experimentation at 48 h postpartum, lasting ~6–9 d (~7 d), the variability depending upon the length of gestation that ranged from 18 to 21 d). At day 14 of pregnancy, mice were randomly assigned to a treatment group and provided with standard chow or chow containing SGE-516 (450 mg/kg chow) or clobazam (250 mg/kg chow) until the time of testing. These doses were chosen because they result in plasma concentrations previously shown to exert centrally-acting effects (26).

For the acute drug treatments, mice were administered either vehicle (5% 2-hydroxypropyl-beta-cyclodextrin [HPβCD]), SGE-516 (5 mg/kg), or clobazam (10 mg/kg). The vehicle used (5% HPβCD) has previously been demonstrated to be inert (27). All drugs were administered by intraperitoneal (*i.p*.) injection 30 min prior to experimentation.

Pharmacokinetic studies were performed to determine the plasma and brain levels reached by both the acute and chronic treatment paradigms. Plasma samples were collected in K2EGTA-coated tubes and centrifuged at 2,000 G for 10 min at 4°C. Whole brains were weighed and snap frozen on dry ice. Plasma and brain samples were stored at−80°C until use. Exposure levels in the tissue and plasma were determined by Pharmacadence (Hatfield, PA). The plasma and brain exposure levels are provided in Table 1. The amount of chow consumed (standard chow: 6.7 ± 0.9 g/day; SGE-516 chow: 6.0 ±0.4 g/day) was not different between treatment groups (data not shown). No signs of sedation or overt changes in health were observed in any of the treatment groups. Homecage locomotor activity was not altered in SGE-516 treated dams (2167.3 ± 75.8 cm/day) compared to standard chow treatment (2351.6 ± 346.6 cm/day). The higher exposure levels following the acute SGE-516 treatment is likely due to the likely due to the route of administration. In the chronic SGE-516 treatment, the mice consume the chow largely during the dark period with levels peaking during this phase. The tissue and plasma were collected in the morning and may not reflect peak exposure values in the chronic SGE-516-treated group; whereas, the acute SGE-516 treatment was performed 30 min prior to sample collection, likely contributing to the higher levels observed following the acute treatment.

**Table 1:** Pharmacological treatments and exposure levies.

**Experiment**	**Dose**	**Route of administration**	**Plasma levels (ng/ml)**	**Brain levels (ng/g)**	**n numbers**
Vehicle	standard chow	oral (chow)	nd	nd	2
Chronic SGE-516	450 mg/kg chow	oral (chow)	72.5 ± 17.4	86.6 ± 211	4
Chronic clobazam	250 mg/kg chow	oral (chow)	54.3 ± 9.9	73.1 ± 11.9	9
Vehicle	5%HPβCD	i.p.	nd	nd	2
Acute SGE-516	5mg/kg	i.p.	415.0 ± 174.8	669.8 ± 265.4	4
Arute Clobazam	10mg/kg	i.p.	94.1 ± 18.6	180.6 ± 27.9	5

*i.p., Intraperitoneally; nd, not detected*.

To assess whether SGE-516 can alter KCC2 expression in the PVN using Western blot analysis, we employed virgin adult female mice (60–90 d of age) to prevent normal peripartum hormonal changes from confounding the results. Virgin females were maintained on either standard chow or SGE-516 chow (450 mg/kg chow) for 18 consecutive days to mimic the elevated levels of neurosteroids throughout pregnancy.

### Behavioral tests

Behavioral tests were conducted on postpartum WT, *Gabrd*^−/−^, *Gabrd*^±/−−^, and KCC2/Crh mice maintained on standard, SGE-516, or clobazam chow or acutely administered vehicle, SGE-516, or clobazam 30 min prior to testing. Animals were subjected to both the forced swim test and the maternal approach test. All behavioral tests occurred between 09:00 and 16:00 h, following at least 1 h of habituation in the behavioral testing room. The behavioral tests were videotaped and scored by two different investigators, one blinded to the experimental condition. This approach was utilized to prevent subjective interpretation of the data. There was no significant difference in the results of the independent scoring; therefore, the analysis from the blinded investigator was used for the final data sets.

The forced swim was conducted as previously described (10, 16, 20). Mice were individually placed into a plastic beaker (21 cm diameter) containing 15 cm of room temperature water (23–25°C). The latency to immobility and the total time spent immobile was measured over the 6 min test. Immobility was considered to be floating with no front paw movement and no movement or minimal movement of a single hind paw. In contrast, directional swimming and active struggling were not included in the time spent immobile. Following the forced swim test, mice were towel dried and placed back into their homecage.

The maternal approach test was performed following the forced swim test as previously described to investigate the impact of stress during the postpartum period on maternal behavior (20). The dams were introduced into their homecage at the opposite corner from their litter after a brief separation (10 min). The latency to approach the pups and the total time spent in contact with pups were measured as indices of maternal behavior. Only >5 s interactions were included in the time spent with the pups. Burrowing beneath or trampling the litter was not included in the measure of pup contact time.

### Corticosterone measurements

Peripartum HPA axis abnormalities were assessed by subjecting virgin mice and postpartum mice to an acute restraint stress (30 min) by placing mice individually into a 50 mL Falcon tube modified with breathing holes and comparing corticosterone levels to unstressed homecage controls. Mice subjected to the 30 min restraint stress or minimally handled controls were anesthetized with isoflurane, rapidly decapitated, trunk blood was collected in CAPIJECT^®;^ (T-MG) tubes, and serum collected by centrifugation for corticosterone measurements. All samples were collected between 10:00 and 12:00 h. Corticosterone was measured as previously described (20, 28, 29), using a commercially available enzyme immunoassay kit, according to the manufacturer's instructions (Enzo Pharmaceuticals, New Jersey). Briefly, samples were run in duplicate and compared to a standard curve of known corticosterone concentrations.

### Western blot

Western blots were carried out as previously described (19, 20, 29, 30). Mice were anesthetized between 10:00 and 12:00 h with isoflurane, sacrificed by guillotine-assisted decapitation, and the PVN was microdissected and placed in ice-cold homogenization buffer (10 mM NaPO4, 100 mM NaCl, 10 mM Na pyrophosphate, 25 mM NaF, 5 mM EDTA, 5 mM EGTA, 2% Triton X-100, 0.5% Deoxycholate, 1 mM Na vanadate, pH 7.4), in the presence of protease inhibitors (complete mini, Roche, in fresh 100 mM PMSF dissolved in ethanol). Total protein was isolated and concentrations were determined using DC Protein Assay (Bio-Rad, Hercules, CA). Total protein (25 μg) was loaded onto a 12% SDS-polyacrylamide gel, subjected to electrophoresis and transferred to a PDVF membrane (Immobilon P, Millipore, Temecula, CA), blocked in 10% nonfat milk, and probed with a polyclonal antibody specific for KCC2 (1:1,000, Millipore, Temecula, CA), a phospho-specific antibody for phosphorylation of KCC2 at residue Ser940 (1:1,000, a generous gift from Dr. Stephen J. Moss), or a monoclonal β-tubulin antibody (1:10,000, Sigma Aldrich, St. Louis, MO). The blots were then incubated with either peroxidase labeled anti-rabbit IgG (1:2,500, GE Healthcare) or peroxidase labeled anti-mouse IgG (1:2,500, GE Healthcare) and immunoreactive proteins were visualized using enhanced chemiluminescence (Amersham/GE Healthcare). All experimental groups were run in parallel. Optical density measurements were performed using NIH ImageJ software and normalized to total protein levels (25 μg total protein) rather than a housekeeping protein, which have shown variability in expression levels (31).

### Statistical analyses

All data were analyzed using GraphPad Prism 6 or Excel. Statistical significance between vehicle and SGE-516 treatment groups for behavioral tests was determined using an unpaired Student's *t*-test. A Mann–Whitney non-parametric test was also performed to verify statistical significance. For all behavioral tests, ANOVAs were not performed to compare genotype and drug because the preclinical PPD models are maintained on different backgrounds and therefore direct comparisons are not appropriate. Further, the vehicles for the drugs are also different, requiring comparison to their respective vehicle controls. Thus, the comparison focused on the effect of drug vs. vehicle, in which a *t*-test is appropriate. Comparison between stress status (control and stress) and treatment (standard chow vs. SGE-516) for the Western blot experiments, on the other hand, was conducted using a two-way ANOVA with a Sidak *post-hoc* test for multiple comparisons. All data are represented as the average + SEM. Statistical significance was defined as *p* < 0.05.

## Results

### Chronic SGE-516 treatment decreases depression-like behaviors in preclinical PPD models

**Figure 1:**
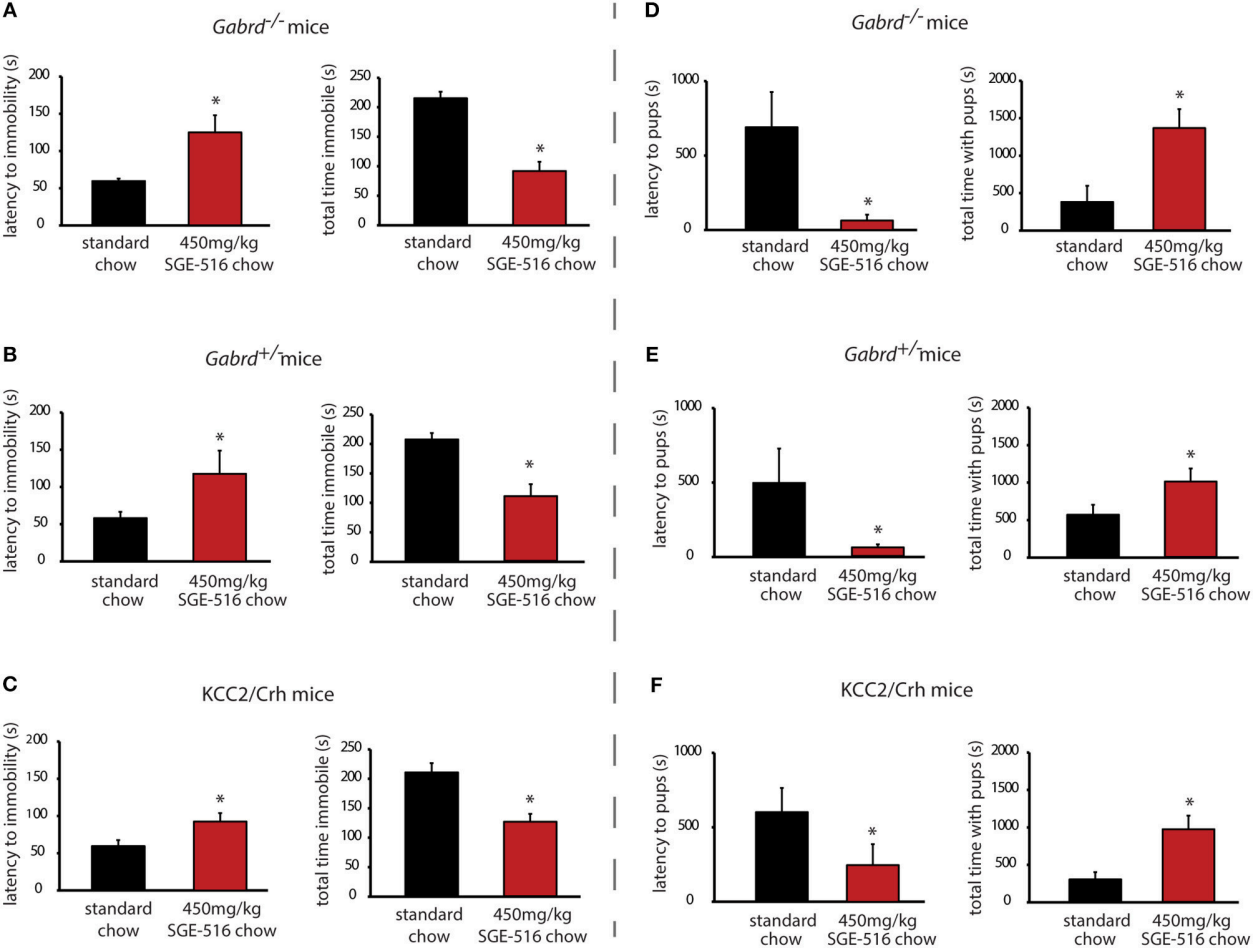
Chronic SGE-516 treatment decreases depression-like behaviors and improves maternal care in preclinical PPD models. Chronic treatment with SGE-516 (450 mg/kg chow, ~7 d) increases the average latency to immobility and decreases the total time spent immobile in postpartum *Gabrd*^−/−^ **(A)**, *Gabrd*^+/−^ **(B)**, and KCC2/Crh dams **(C)** in the forced swim test. *Denotes statistical significance of *p* < 0.05 using a Student's *t*-test. Chronic treatment with SGE-516 (450 mg/kg chow, ~7 d) decreases the average latency to approach their pups and increases to total interaction time with the pups in postpartum *Gabrd*^−/−^ **(D)**, *Gabrd*^+/−^ **(E)**, and KCC2/Crh dams **(F)** in the maternal approach test. *Denotes statistical significance of *p* < 0.05 using a Student's *t*-test.

*Gabrd*^−/−^, *Gabrd*^+/−^, and KCC2/Crh mice exhibit depression-like behaviors during the postpartum period compared to their respective wild type controls (Table 2) (10, 16, 20). *Gabrd*^−/−^, *Gabrd*^+/−^, and KCC2/Crh mice exhibit a decrease in the latency to immobility and an increase in the total time spent immobile in the forced swim test compared to wild type at 48 h postpartum (Table 2; *p* < 0.05 using a Student's *t*-test compared to wild type). Here we demonstrate that chronic treatment of these preclinical PPD models with SGE-516 decreases depression-like behaviors during the postpartum period. *Gabrd*^−/−^ dams treated with SGE-516 exhibit an increase in the latency to the first bout of immobility and a decrease in the total time immobile compared to standard chow-treated *Gabrd*^−/−^ dams [Figure 1A, Table 2; *p* < 0.05 using a Student's *t*-test; latency: *t*_(17)_ = −2.97; total time immobile; *t*_(17)_ = 6.59]. Similarly, chronic treatment of *Gabrd*^+/−^ dams with SGE-516 results in an increase the latency to immobility and a decrease in the total time immobile in the forced swim test compared to standard chow-treated *Gabrd*^+/−^ dams [Figure 1B, Table 2; *p* < 0.05 using a Student's *t*-test; latency: *t*_(20)_ = −2.01; total time immobile; *t*_(20)_ = 4.60]. Mice lacking KCC2 in CRH neurons (KCC2/Crh) also exhibit depression-like behaviors restricted to the postpartum period that improves with SGE-516 treatment. SGE-516 treatment increases the latency to immobility and decreases the total time spent immobile in KCC2/Crh dams in the forced swim test compared to their standard chow-treated counterparts [Figure 1C, Table 2; *p* < 0.05 using a Student's *t*-test; latency: *t*_(25)_ = −2.29; total time immobile; *t*_(25)_ = 4.10]. It is important to note that SGE-516 treatment does not alter behavior in the forced swim test in postpartum wild type mice (latency: 91.3 ± 3.8 s; total time immobile: 119.8 ± 14.2 s) compared to standard chow-treated wild type dams (latency: 82.2 ± 7.9 s; total time immobile: 131.3 ± 14.3 s) [data not shown; *p* > 0.05 using a Student's *t*-test; latency: *t*_(11)_ = −0.73; total time immobile; *t*_(11)_ = 0.49].

**Table 2:** Acute and Chronic SGE-516 treatment improves abnormal postpartum behaviors in two preclinical PPD models.

**Genotype**	**Treatment**	**Latency to immobility**	**Total time immobile**	***n***	**Latency to approach**	**Total interaction time**	***n***
Wild Type	Standard Chow	82.2 ± 7.9 s	131.3 ± 14.3 s	9	54.2 ± 12.6 s	1408.2 ± 187.2 s	8
Gabrd^−/−^dams	Standard Chow	59.5 ± 3.2 s^#^	215.7 ± 11.1 s^#^	10	697.5 ± 197.3 s^#^	429.7 ± 177.8 s^#^	10
Gabrd^−/−^dams	Chronic SGE-516 (450 mg/kg chow)	124.8 ± 23.0 s^*^	92.3 ± 15.4 s^*^	9	58.6 ± 33.0 s^*^	1411.9 ± 219.3 s	7
Gabrd^−/−^dams	Standard Chow	54.3 ± 8.6 s^#^	227.6 ± 18.7 s^#^	7	752.9 ± 167.7 s^#^	376.4 ± 151.2 s^#^	12
Gabrd^−/−^dams	Chronic Clobazam (250 mg/kg chow)	54.4 ± 6.9 s	236.9 ± 8.3 s	9	405.0 ± 143.6 s	133.6 ± 34.1 s	7
Gabrd^+/−^dams	Standard Chow	57.5 ± 7.8 s^#^	207.1 ± 10.4 s^#^	12	580.5 ± 226.7 s^#^	529.5 ± 125.8 s^#^	11
Gabrd^+/−^dams	Chronic SGE-516 (450 mg/kg chow)	112.0 ± 28.4 s^*^	113.5 ± 18.5 s^*^	10	57.8 ± 17.6 s^*^	1081.0 ± 168.4 s^*^	9
KCC2/Crh	Standard Chow	59.5 ± 8.1 s^#^	207.8 ± 15.3 s^#^	10	602.1 ± 162.5 s^#^	301.0 ± 96.4 s^#^	14
KCC2/Crh	Chronic SGE-516 (450 mg/kg chow)	95.9 ± 11.2 s^*^	125.7 ± 12.4 s^*^	17	229.2 ± 129.9 s^*^	973.9 ± 169.6 s^*^	13
KCC2/Crh	Standard Chow	54.5 ± 7.6 s^#^	217.8 ± 15.2 s^#^	10	655.5 ± 147.7 s^#^	310.9 ± 86.1 s^#^	11
KCC2/Crh	Chronic Clobazam (250 mg/kg chow)	40.0 ± 6.3 s	241.1 ± 7.2 s	8	801.4 ± 223.6 s	310.7 ± 127.6 s	7
Gabrd^−/−^dams	Vehicle	62.8 ± 5.7 s	233.4 ± 18.2	9	901.4 ± 195.3 s	358.4 ± 141.3 s	11
Gabrd^−/−^dams	Acute SGE-516 (5 mg/kg)	85.4 ± 4.9 s^*^	175.0 ± 15.7 s^*^	12	143.3 ± 96.2 s^*^	725.0 ± 166.8 s^*^	9
Gabrd^−/−^dams	Vehicle	56.3 ± 8.1 s	218.5 ± 15.6 s	8	774.4 ± 207.1 s	317.1 ± 163.3 s	8
Gabrd ^−/−^dams	Acute Clobazam (10 mg/kg)	59.4 ± 5.0 s	253.4 ± 5.0 s	9	1143.5 ± 242.8 s	152.0 ± 73.8 s	10
KCC2/Crh	Vehicle	60.9 ± 6.9 s	221.2 ± 15.2 s	11	791.3 ± 191.2 s	232.3 ± 94.2 s	16
KCC2/Crh	Acute SGE-516 (5 mg/kg)	94.5 ± 9.5 s^*^	154.2 ± 17.0 s^*^	11	108.3 ± 66.1 s^*^	723.1 ± 129.6 s^*^	9
KCC2/Crh	Vehicle	61.9 ± 9.2 s	206.0 ± 18.3 s	8	884.3 ± 249.2 s	258.5 ± 139.3 s	10
KCC2/Crh	Acute Clobazam (10 mg/kg)	55.5 ± 9.3 s	252.7 ± 9.2 s	10	806.9 ± 206.1 s	398.8 ± 190.3 s	8

# *Denotes p < 0.05 compared to Wild Type mice treated with vehicle or standard chow*;

* *Denotes p < 0.05 compared to vehicle or standard chow treated mice within genotype*.

### Chronic SGE-516 treatment improves deficits in maternal care in preclinical PPD models

*Gabrd*^−/−^, *Gabrd*^+/−^, and KCC2/Crh mice exhibit deficits in maternal care compared to wild type dams (Table 2) (10, 20). *Gabrd*^−/−^, *Gabrd*^+/−^, and KCC2/Crh mice exhibit an increase in the latency to approach their pups and exhibit a decrease in the total time interacting with their pups in the maternal approach test compared to their respective wild types (Table 2; *p* < 0.05 using a Student's *t*-test compared to wild type). SGE-516 treatment improves maternal care in preclinical PPD models. Chronic treatment of *Gabrd*^−/−^ mice with SGE-516 decreases the latency to approach and increases the total time interacting with their pups in the maternal approach test compared to standard chow-treated *Gabrd*^−/−^ dams [Figure 1D, Table 2; *p* < 0.05 using a Student's *t*-test; latency to approach: *t*_(15)_ = 2.67; total interaction time; *t*_(15)_ = −3.50]. Similarly, treatment of *Gabrd*^+/−^ dams with SGE-516 decreases the latency to approach and increases the total interaction time with their pups compared to standard chow-treated *Gabrd*^+/−^ dams [Figure 1E, Table 2; *p* < 0.05 using a Student's *t*-test; latency to approach: *t*_(18)_ = 2.07; total interaction time; *t*_(18)_ = −2.68). Maternal care in KCC2/Crh dams is also improved with SGE-516 treatment, with a decrease in the latency to approach their pups and a significant increase in the total interaction time compared to compared to standard chow-treated postpartum KCC2/Crh mice [Figure 1F, Table 2; *p* < 0.05 using a Student's *t*-test; latency to approach: *t*_(27)_ = 1.73; total interaction time; *t*_(27)_ = −3.51]. However, SGE-516 treatment does not alter maternal care measured using the maternal approach test in postpartum wild type mice (latency: 503.0 ± 286.3 s; total interaction time: 1292.0 ± 232.6 s) compared to standard chow-treated wild type dams (latency: 490.6 ± 285.9 s; total interaction time: 1056.1 ± 268.1 s) [data not shown; *p* > 0.05 using a Student's *t*-test; latency: *t*_(11)_ = −0.03; total time immobile; *t*_(11)_ = −0.61].

### SGE-516 suppresses the stress-induced activation of the HPA axis

**Figure 2:**
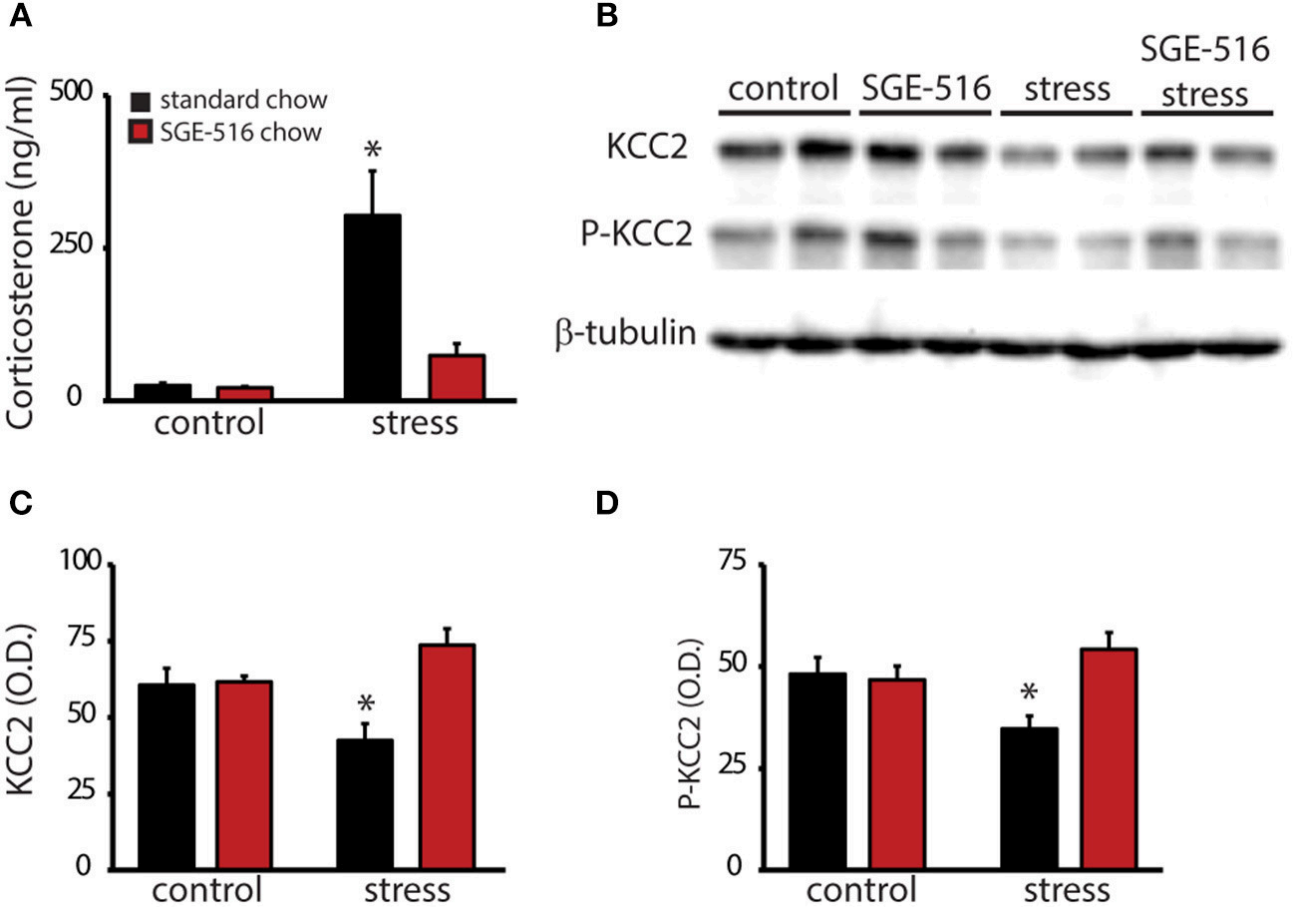
SGE-516 mimics suppression of the HPA axis during the peripartum period. **(A)** The average baseline and stress-induced circulating corticosterone levels in virgin mice treated with standard or SGE-516 chow (450 mg/kg chow, 18 d). Stress-induced elevations in corticosterone are decreased in mice treated with SGE-516 compared to standard chow-treated mice. **(B)** Representative Western blots of total protein isolated from the PVN of virgin mice maintained on either standard or SGE-516 chow and subjected to a 30 min restraint stress or minimally-handled controls and probed with an antibody specific for KCC2, phosphorylated KCC2 at residue Ser940 (P-KCC2), or β-tubulin. The average optical density of KCC2 **(C)** and P-KCC2 **(D)** expression is decreased following stress in mice maintained on standard chow but not in mice maintained on SGE-516 chow. *Denotes statistical significance of *p* < 0.05 using a two-way ANOVA with a Sidak *post-hoc* test for multiple comparisons.

To investigate whether the therapeutic effects of SGE-516 may involve regulation of the HPA axis, virgin wild type mice were treated with either standard chow or SGE-516 (18 d treatment) and baseline and stress-induced corticosterone levels were measured. Baseline corticosterone levels are not different between standard chow (25.0 ± 4.5 ng/ml) or SGE-516 (21.3 ± 2.6 ng/ml) treated mice. However, following a single, 30 min restraint stress, corticosterone levels are decreased in SGE-516-treated mice (74.2 ± 19.7 ng/ml) compared to standard chow-treated mice (303.2 ± 73.3 ng/ml) (Figure 2A; *n* = 9–11 mice per experimental group). There is a significant interaction between treatment and stress status in corticosterone levels [*p* < 0.05 using a two-way ANOVA; *F*_(1, 36)_ = 10.83].

To examine whether SGE-516 suppresses the stress-induced activation of the HPA axis involving a KCC2-dependent mechanism, we examined KCC2 expression in the PVN in standard chow- and SGE-516-treated mice in unstressed, minimally handled controls and in mice subjected to a 30 min restraint stress. As previously demonstrated, acute restraint stress decreases the phosphorylation of KCC2 at residue Ser940 (34.5 ± 3.2 O.D. units/25 μg total protein) and decreases total KCC2 expression in the PVN (42.2 ± 5.6 O.D. units/25 μg total protein) compared to minimally handled controls (P-KCC2: 48.0 ± 4.1 O.D. units/25 μg total protein; KCC2: 60.4 ± 5.5 O.D. units/25 μg total protein) (Figures 2B–D). SGE-516 treatment prevents the stress-induced dephosphorylation of KCC2 at residue Ser940 (54.1 ± 4.1 O.D. units/25 μg total protein) and the downregulation of KCC2 expression in the PVN (73.5 ± 5.4 O.D. units/25 μg total protein) compared to minimally handled SGE-treated mice (P-KCC2: 46.6 ± 3.4 O.D. units/25 μg total protein; KCC2: 61.4 ± 1.9 O.D. units/25 μg total protein) and minimally handled, standard chow-treated controls (P-KCC2: 48.0 ± 4.1 O.D. units/25 μg total protein; KCC2: 60.4 ± 5.5 O.D. units/25 μg total protein) (Figures 2B–D). There is a significant interaction between treatment and stress status in the phosphorylation of KCC2 at residue Ser940 [*p* < 0.05 using a two-way ANOVA; *F*_(1, 36)_ = 7.796] and total KCC2 expression [*p* < 0.05 using a two-way ANOVA; *F*_(1, 36)_ = 9.707).

### Chronic clobazam treatment is ineffective at altering the depression-like phenotype in preclinical PPD models

**Figure 3:**
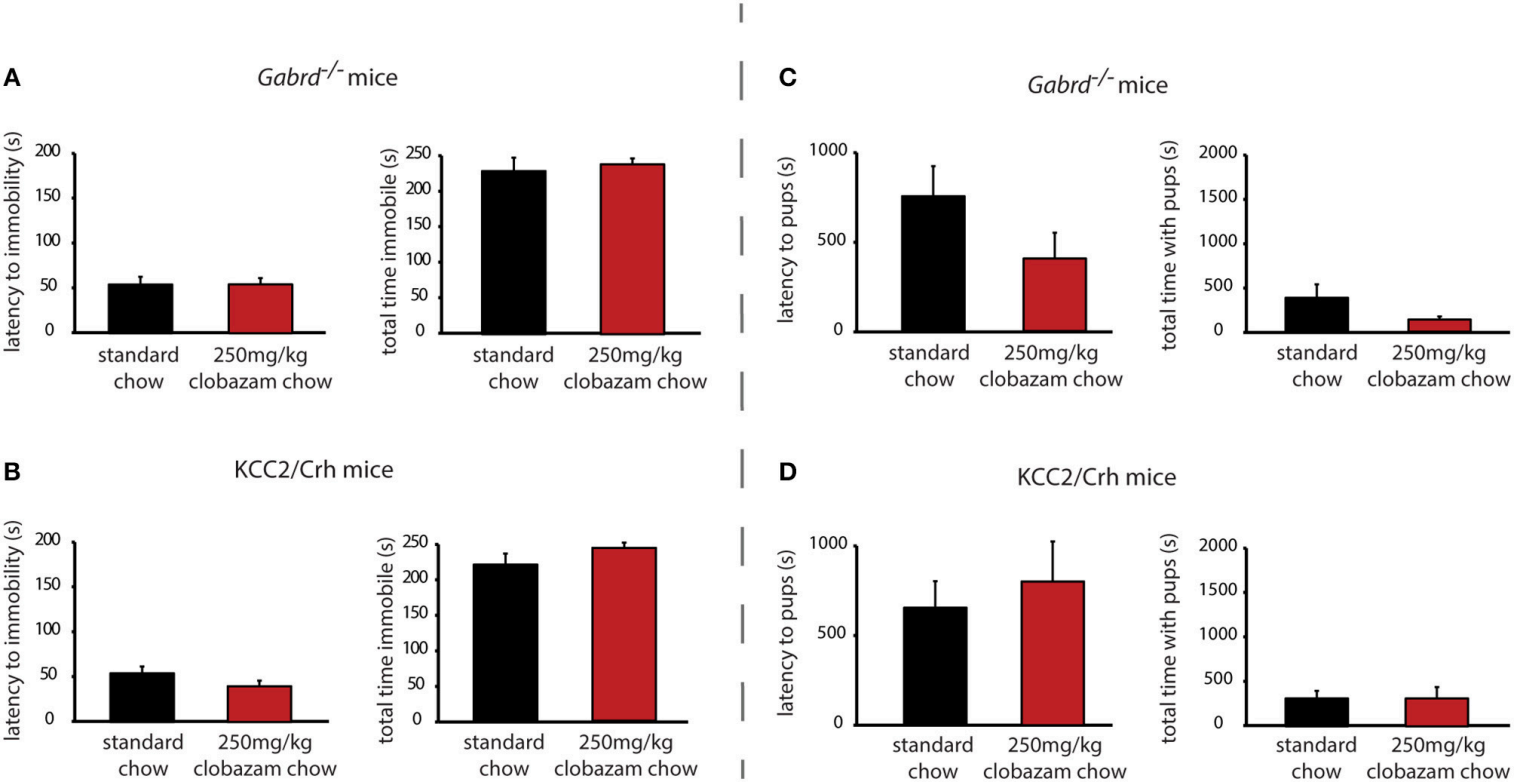
Chronic clobazam is ineffective at altering depression-like behaviors or improving maternal care in preclinical PPD models. Treatment with clobazam (250 mg/kg chow, ~7 d) does not alter the average latency to immobility or the total time spent immobile in postpartum *Gabrd*^−/−^ **(A)** or KCC2/Crh dams **(B)** in the forced swim test. Treatment with clobazam (250 mg/kg chow, ~7 d) does not alter the average latency to approach or the total pup interaction time in postpartum *Gabrd*^−/−^ **(C)** or KCC2/Crh dams **(D)** in the maternal approach test.

In contrast to SGE-516, the benzodiazepine, Clobazam, is ineffective at altering depression-like behaviors in postpartum *Gabrd*^−/−^ or KCC2/Crh mice. Chronic clobazam treated *Gabrd*^−/−^ mice exhibit a similar latency to immobility and total time spent immobile in the forced swim test compared to standard chow-treated *Gabrd*^−/−^ dams [Figure 3A, Table 2; ns determined using a Student's *t*-test; latency: *t*_(14)_ = −0.01; total time immobile; *t*_(14)_ = −0.49].

Similarly, clobazam is ineffective at reducing depression-like behaviors in postpartum KCC2/Crh mice. The latency to immobility is similar between clobazam and standard chow-treated KCC2/Crh dams and the total time immobile is not significantly different between these treatment groups [Figure 3B, Table 2; ns determined using a Student's *t*-test; latency: *t*_(16)_ = 1.42; total time immobile; *t*_(16)_ = −1.27].

### Chronic clobazam treatment is ineffective at altering the deficits in maternal care in preclinical PPD models

Unlike SGE-516, clobazam is also ineffective at improving maternal care in *Gabrd*^−/−^ and KCC2/Crh dams. The latency to approach their pups in the maternal approach test is not affected by clobazam treatment compared to standard chow-treated controls [Figure 3C, Table 2; ns determined using a Student's *t*-test; *Gabrd*^−/−^: *t*_(17)_ = 1.41; KCC2/Crh: *t*_(16)_ = −0.57]. The total interaction time in the maternal approach test is also not significantly different between clobazam-treated KCC2/Crh dams and standard chow-treated controls [Figure 3D, Table 2; ns determined using a Student's *t*-test; *Gabrd*^−/−^: *t*_(17)_ = 1.20; KCC2/Crh: *t*_(16)_ = 0.01].

### Acute SGE-516 treatment improves the depression-like behaviors in preclinical PPD models

**Figure 4:**
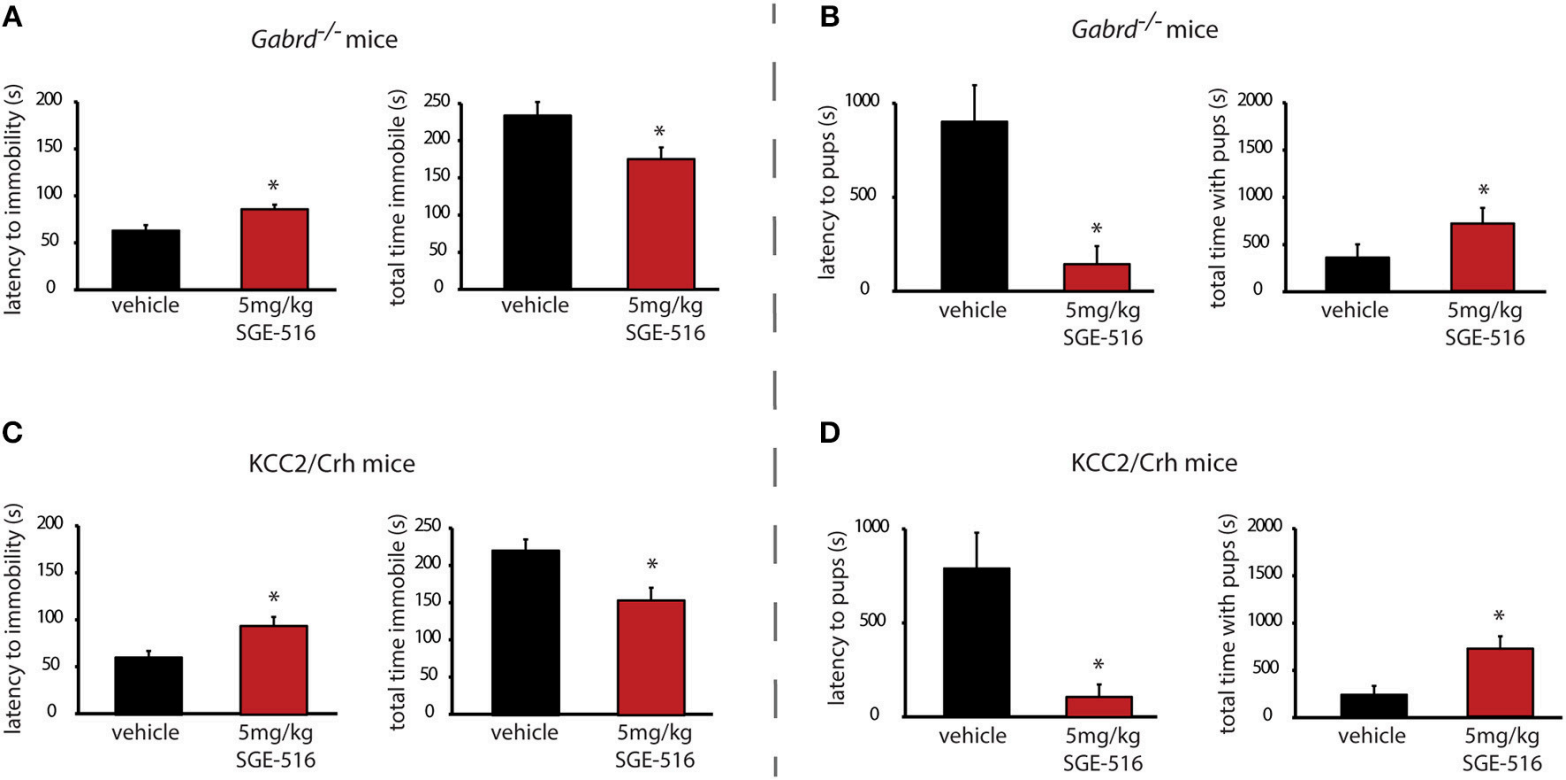
Acute SGE-516 treatment decreases depression-like behaviors and improves maternal care in preclinical PPD models. Acute treatment with SGE-516 (5 mg/kg, 30 min prior to testing) increases the average latency to immobility and decreases the total time spent immobile in postpartum *Gabrd*^−/−^ **(A)** and KCC2/Crh dams **(B)** in the forced swim test. *Denotes statistical significance of *p* < 0.05 using a Student's *t*-test. Acute treatment with SGE-516 (5 mg/kg, 30 min prior to testing) decreases the average latency to approach their pups and increases to total interaction time with the pups in postpartum *Gabrd*^−/−^ **(C)** and KCC2/Crh dams **(D)** in the maternal approach test. *Denotes statistical significance of *p* < 0.05 using a Student's *t*-test.

To further explore the time course of the therapeutic effects of SGE-516, we examined the ability of acute SGE-516 treatment to alleviate depression-like behaviors in preclinical PPD models. Acute SGE-516 treatment (5 mg/kg, *i.p*.) decreased depression-like behaviors during the postpartum period in both *Gabrd*^−/−^ and KCC2/Crh mice. The latency to the first bout of immobility in the forced swim test is increased and the total time spent immobile is decreased in acute SGE-516-treated *Gabrd*^−/−^ dams compared to vehicle treated mice [Figure 4A, Table 2; *p* < 0.05 using a Student's *I*-test; latency: *t*_(19)_ = -3.03; total time immobile; *t*_(19)_ = 2.43]. Similarly, acute SGE-516 treatment in KCC2/Crh dams increases the latency to immobility and decreases the total time spent immobile in the forced swim test compared to vehicle-treated KCC2/Crh dams [Figure 4B, Table 2; *p* < 0.05 using a Student's *t*-test; latency: *t*_(20)_ = −2.86; total time immobile; *t*_(20)_ = 2.93].

### Acute SGE-516 treatment improves the deficits in maternal care in preclinical PPD models

Acute SGE-516 treatment also improves maternal care in *Gabrd*^−/−^ and KCC2/Crh dams. The latency to approach their pups is decreased and the total interaction time in the maternal approach test is increased in acute SGE-516 treated *Gabrd*^−/−^ and KCC2/Crh dams compared to vehicle-treated controls [Figures 4C,D, Table 2; *p* < 0.05 using a Student's *t*-test; *Gabrd*^−/−^: latency: *t*_(18)_ = 3.25; total interaction time; *t*_(18)_ = −1.68; KCC2/Crh: latency: *t*_(23)_ = 2.61; total interaction time; *t*_(23)_ = −3.12].

### Acute clobazam treatment does not alter the depression-like phenotype in Gabrd^−/−^ and KCC2/Crh mice

**Figure 5:**
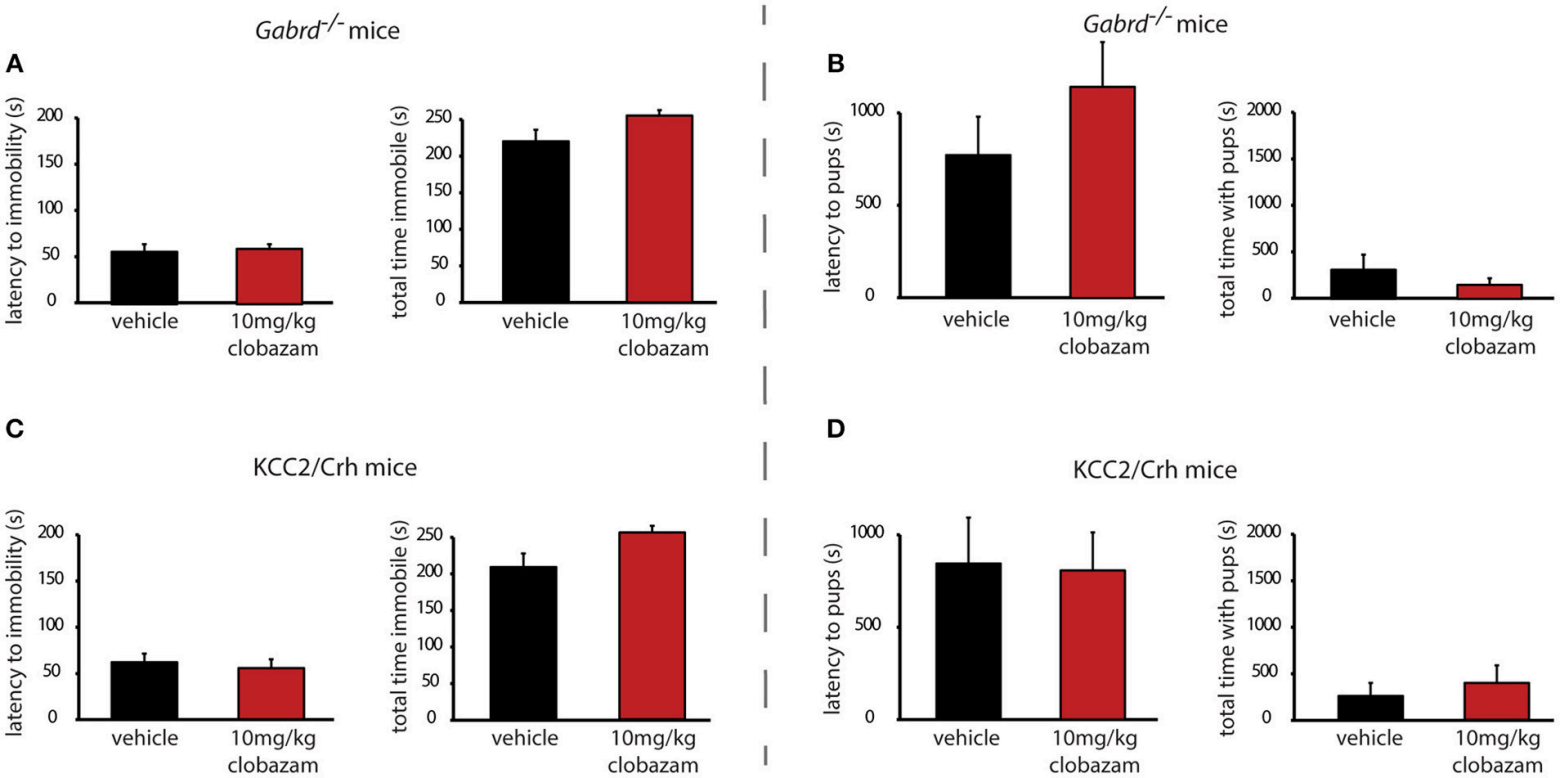
Acute clobazam is ineffective at altering depression-like behaviors or improving maternal care in preclinical PPD models. Acute clobazam treatment (10 mg/kg chow, 30 min prior to testing) does not alter the average latency to immobility or the total time spent immobile in postpartum *Gabrd*^−/−^ **(A)** or KCC2/Crh dams **(B)** in the forced swim test. Acute treatment with clobazam (10 mg/kg chow, 30 min prior to testing) does not alter the average latency to approach or the total pup interaction time in postpartum *Gabrd*^−/−^ **(C)** or KCC2/Crh dams **(D)** in the maternal approach test.

Similar to the results obtained with chronic clobazam treatment, acute clobazam treatment is also ineffective at altering the depression-like phenotype observed during the postpartum period in preclinical PPD models. There is no difference in the latency to immobility in the forced swim test in acute clobazam-treated *Gabrd*^−/−^ or KCC2/Crh dams compared to vehicle-treated controls [Figures 5A,B, Table 2; ns determined using a Student's *t*-test; *Gabrd*^−/−^: *t*_(15)_ = −0.34; KCC2/Crh: *t*_(16)_ = 0.48]. Similarly, acute clobazam treatment does not alter the total time spent immobile in the forced swim test in *Gabrd*^−/−^ or KCC2/Crh dams compared to vehicle-treated controls [Figures 5A,B, Table 2; ns determined using a Student's *t*-test; *Gabrd*^−/−^: *t*_(15)_ = −2.11; KCC2/Crh: *t*_(16)_ = −2.42].

### Acute clobazam treatment does not alter the deficits in maternal care in Gabrd^−/−^ and KCC2/Crh mice

Again, similar to the results obtained with chronic clobazam treatment, acute clobazam treatment does not alter the deficits in maternal care observed in preclinical PPD models. Acute clobazam treatment in *Gabrd*^−/−^ dams does not alter the latency to approach or the total interaction time with their pups in the maternal approach test compared to vehicle-treated *Gabrd*^−/−^ dams [Figure 5C, Table 2; ns determined using a Student's *t*-test; latency to approach: *t*_(16)_ = 1.73; total interaction time: *t*_(16)_ = 0.99]. Clobazam treatment in KCC2/Crh mice also does not change the latency to approach their pups or total pup interaction time in the maternal approach test compared to vehicle-treated KCC2/Crh dams [Figure 5D, Table 2; ns determined using a Student's *t*-test; latency to approach: *t*_(16)_ = 0.11; total interaction time: *t*_(16)_ = −0.59].

## Discussion

Here we demonstrate that U treatment with SGE-516 is effective at ameliorating abnormal postpartum behaviors in preclinical PPD models, decreasing depression-like behaviors and improving maternal care. The evidence that acute treatment with SGE-516 is effective in preclinical PPD models is translationally important, suggesting potential fast-acting treatment effects, which is consistent with the rapid onset of observed with a similar compound, brexanolone, in clinical trials (21, 22).

These therapeutic effects of SGE-516 in preclinical PPD models are distinct and do not translate to other GABAergic modulators, such as benzodiazepines, given the ineffectiveness of clobazam to alter postpartum depression-like behaviors or improve maternal care. This evidence provides an important distinction that speaks to the therapeutic mechanism of action and implicates neurosteroid-sensitive, extrasynaptic GABA_A_Rs that mediate tonic inhibition, rather than benzodiazepine-sensitive synaptic receptors that mediate the phasic component of GABAergic inhibition.

Interestingly, the therapeutic effects of SGE-516 in *Gabrd*^−/−^dams suggest that the effects of SGE-516 may be mediated by actions on extrasynaptic GABA_A_R subtypes that mediate the residual tonic current in *Gabrd*^−/−^ mice. Further, the PK values suggest that the levels of SGE-516 in the brain are not high enough to activate synaptic GABA_A_Rs. These findings suggest that the therapeutic effects of SGE-516 are distinct and not shared with benzodiazepines.

In addition to the direct allosteric modulation of GABA_A_Rs, neurosteroids have also been shown to regulate GABA_A_Rs via post-translational modifications, such as phosphorylation-dependent trafficking of receptors that are in part dependent upon protein kinase C [for review see (32)]. For example, allopregnanolone and Tetrahydrodeoxycorticosterone (THDOC) have been demonstrated to increase the surface expression of GABA_A_Rs via a PKC-dependent mechanism, resulting in a sustained increase in the efficacy of GABAergic inhibition, through phosphorylation of the GABA_A_R α4 subunit at residue Ser443 (S443) and the GABA_A_R β3 subunit at Serine residues 408 and 409 (S408/9) [for review see (32)]. Therefore, SGE-516 may facilitate GABAergic inhibition independent of or in addition to the direct allosteric potentiation of GABA_A_R δ subunit-containing receptors, which are uniquely sensitive to neurosteroid modulation (9–12) [for review see (13, 14)]. In fact, SGE-516 has been demonstrated to induce a sustained increased in tonic GABAergic inhibition, which is associated with an increase in the phosphorylation and surface expression of the β3 subunit-containing GABA_A_Rs (33). Interestingly, although ganaxolone was demonstrated to effectively allosterically modulate GABA_A_Rs, it does not exert the sustained increase in tonic GABAergic inhibition observed with the SGE-516 compound (33). Thus, it is possible that the therapeutic effects of SGE-516 involve metabotropic regulation of GABA_A_Rs; however, further studies are required to determine whether this mechanism underlies the therapeutic effects of SGE-516 in preclinical PPD models.

In addition, our previous studies implicated HPA axis dysfunction in contributing to the abnormal postpartum phenotype in both *Gabrd*^−/−^ and KCC2/Crh mice (10, 20). Data presented here demonstrate that SGE-516 is capable of suppressing the stress-induced activation of the HPA axis. However, we still do not fully understand how the HPA axis is regulated during the peripartum period and, therefore, the impact of SGE-516 in the regulation of the HPA axis during this period is also unclear. The current study demonstrates that SGE-516 treatment suppresses the stress-induced activation of the HPA axis via maintenance of KCC2 phosphorylation at residue Ser940 and total KCC2 expression in the PVN, suggesting a potential role in the peripartum regulation of the HPA axis. Previous studies implicate neurosteroids in the suppression of the HPA axis during the postpartum period (34–36) and are thought to involve a GABAergic mechanism (34, 37). We recently demonstrated that the suppression of the stress-induced activation of the HPA axis during the peripartum period involves a KCC2-dependent mechanism (20). The stress-induced dephosphorylation of KCC2 at residue Ser940 and downregulation of KCC2 in the PVN is prevented during the peripartum period (20), which we propose is critical for peripartum stress hyporeactivity that is an essential neuroendocrine adaptation, reducing vulnerability for maladaptive postpartum behaviors. It remains unclear how KCC2 is regulated in the PVN during the peripartum period, but it is possible that neurosteroids may play a role, which is an active area of ongoing research. In support of this hypothesis, here we demonstrate that SGE-516 treatment is capable of preventing the stress-induced dephosphorylation and downregulation of KCC2 and attenuates the stress-induced elevations in corticosterone levels.

Importantly, we have now established preclinical models of PPD useful for the screening of therapeutic compounds. The findings presented here are important because they demonstrate the therapeutic effectiveness of SGE-516 in several relevant preclinical models of postpartum depression. There are very few well-controlled studies that have compared the effectiveness of different classes of compounds for the treatment of PPD preclinically or clinically [for review see (38, 39)]. This study takes the first steps in comparing the ability of different classes of GABA receptor modulators to improve postpartum behaviors in preclinical models. Patients with postpartum depression are typically treated with either selective serotonin reuptake inhibitors (SSRIs) or serotonin-norepinephrine reuptake inhibitors (SNRIs) and there is no evidence that these treatments target the underlying biology of the disorder. The therapeutic effectiveness of SGE-516 in these preclinical PPD models is consistent with the positive clinical trial results using brexanolone for the treatment of PPD (21, 22). Further, these findings validate the use of these preclinical PPD models for screening and comparison of compounds for the treatment of PPD.

## Author contributions

LM and JM were involved in conducting the experiments and data analysis included in the manuscript. LM, JM, RH, and ML were all involved in study design and interpretation of the results. JM wrote the manuscript with input from LM, RH, and ML.

### Conflict of interest statement

JM serves on the Scientific Advisory Board for SAGE Therapeutics and receives financial support for research related to the current study. RH and ML are both employees of SAGE Therapeutics. The remaining author declares that the research was conducted in the absence of any commercial or financial relationships that could be construed as a potential conflict of interest.
